# Supplementary material for: Metformin impairs trophoblast metabolism and differentiation in a dose-dependent manner
Source: Front Cell Dev Biol. 2023 May 12;11:1167097. doi: 10.3389/fcell.2023.1167097 (PMC10213689; doi:10.3389/fcell.2023.1167097)

Supplemental Figure 1: Metformin impacts extracellular acidification rates

A-C) Normalized extracellular acidification rate for BeWo cells treated with vehicle, 200 μM metformin, or 2000 μM metformin the presence of DMSO (0.4%, vehicle) or 40 μM forsksolin (FSK) for 48 hours. n= 4 independent replicates per condition

D) Basal ECAR for BeWo cells treated with vehicle, 200 μM metformin, or 2000 μM metformin. Data representative of mean +/- SEM. n=4 biologic replicates per condition. *, $p<0.05$; **, $p<0.01$; ***, $p<0.001$; and ****, $p<0.0001$.

Supplemental Figure 1:

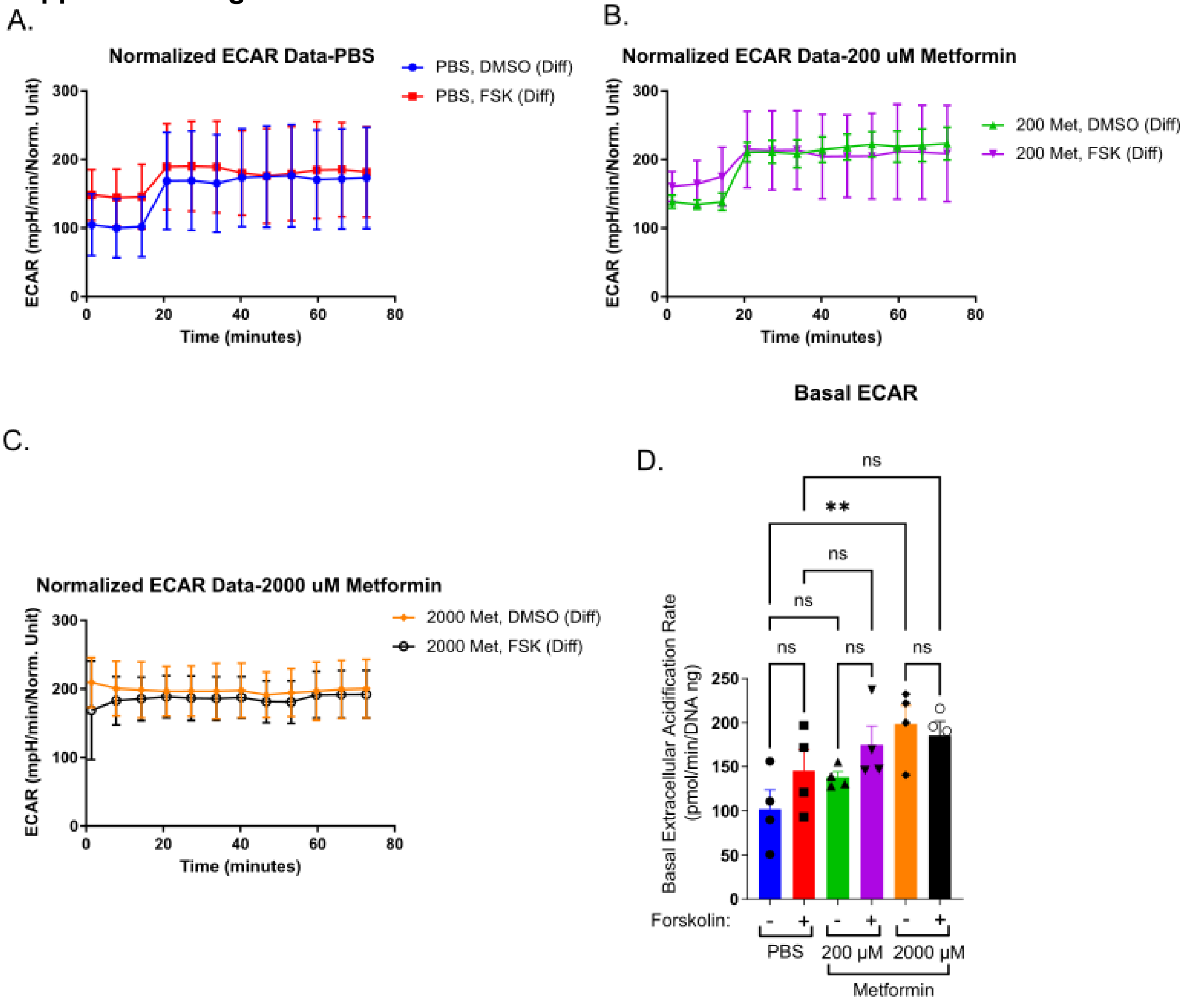

Supplemental Figure 2: Model depicting the impact “therapeutic” and “supra-therapeutic” concentrations of metformin have on trophoblast differentiation and metabolism

Supplemental Figure 2:

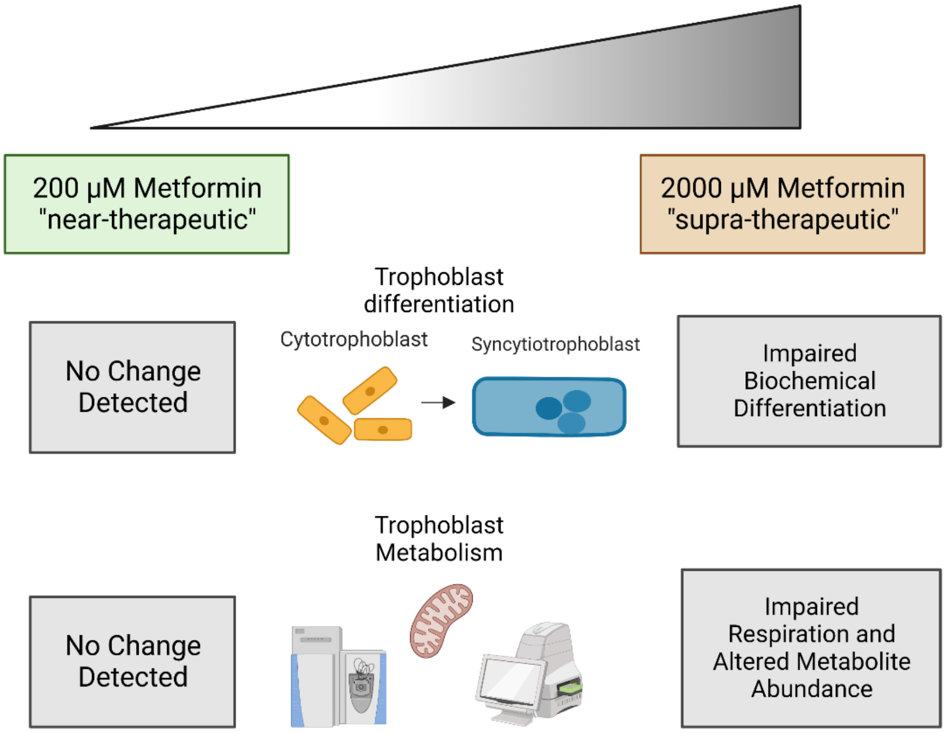

Supplement: Supplementary file 1 [file DataSheet1.PDF]
